# Shifting the Paradigm: A Constructivist Analysis of Agency and Structure in Sustained Youth Sport Participation

**DOI:** 10.3389/fpsyg.2021.660080

**Published:** 2021-04-01

**Authors:** Meredith Flaherty, Michael Sagas

**Affiliations:** Laboratory for Athlete Development and Research, Department of Sport Management, University of Florida, Gainesville, FL, United States

**Keywords:** Structuration Theory, youth sport, participation, attrition, resources, agency, structure

## Abstract

To examine the impact of the relationship between agency and structure on sustained participation in youth sport, semi-structured interviews were conducted with male college soccer players. The participants' accounts (*N* = 20) of their youth careers were analyzed through the lens of Structuration Theory (ST) framed in a constructivist paradigm. ST supports the significance of the recursive relationship between agent and structure in-context in the co-construction of experiences, and provides a framework for analyzing effects of compounding experiences gained across time and space as they influence sport continuation. Clarity of expectations imposed in-context and the athlete's perceived impact on the structure evidenced, through deductive thematic analysis, as the most salient determinants of the perceived valence of the youth sport environment. The agent's perceived holding of authoritative resources across time and contexts was a critical dimension of the participants' continuation in youth sport, substantiating ST as a theoretical lens, situated in a constructivist paradigm, that might add depth to understanding patterns in participation and attrition.

## Introduction

Sport participation during the school age and adolescent child development stages is purported to promote higher self-esteem, higher self-confidence, increased self and social awareness, improved social interaction, and improved physical development and trans-contextual fitness habits (Gould, 2019; Howie et al., 2020). Additionally, sport participation has shown to support the opportunity to build cultural and social capital –antecedents to social mobility (Coakley, 2017; Howie et al., 2020). Despite the overwhelming evidence of the positive impact sport participation might have on the individual, trends in youth sport have evidenced a decline in participation rates and an increase in attrition rates (Balish et al., 2014; Turner et al., 2015; Aspen Institute, 2019).

The decrease in participation rates is a convoluted and multifaceted issue because the foundational constructs present as paradigmatic for researchers—the increased risk of obesity and health-related illness for those who do not participate are quantifiable (Turner et al., 2015; Witt and Dangi, 2018; Gould, 2019); however, the opportunity cost of decreased participation manifests in the loss of psychological, psychosocial, and fitness habit development, which are more difficult constructs to measure (Fraser-Thomas et al., 2005; Turner et al., 2015; Witt and Dangi, 2018; Gould, 2019). Of those populations that have seen the greatest decrease in participation rates (low SES and Black and Latinx children), access and opportunity to participate are identified as limiting factors (Aspen Institute, 2019; Gould, 2019).

Exacerbating the urgency around social issues in youth sport, participation and attrition patterns are of particular concern coming out of an unprecedented pandemic era in which youth sport opportunities might be increasingly consolidated into privatized organizations and structures (Coakley, 2017; Farrey, 2021). While attrition rates have historically been highest in the adolescent years, macro-level research indicates that attrition rates among adolescents have been exacerbated by elevated competitive intensity, exclusivity, and early immersion in youth sport environments (Witt and Dangi, 2018; Aspen Institute, 2019). In contrast, motivation research, generally positioned in a post-positivist research paradigm, has generated a body of knowledge around how a youth sport climate in which the objectives of winning and early success supersede objectives around age-appropriate psychological, psychosocial, and physical development detract from youths' motivation to sustain participation (Petitpas et al., 2005; Wendling et al., 2018; Bateman et al., 2020).

The rich body of knowledge on youth sport participation patterns is overwhelmingly situated in a post-positivist paradigm in which motivation is purported as the principal determinant of sustained participation and where frameworks designed to support findings about motivation operate in a context-specific snapshot (Balaguer et al., 2017; Bateman et al., 2020). In order develop novel insights and potential research veins to disrupt the reliably bleak youth sport participation trends, participation and the processes of continuation is framed here through a constructivist lens, which operates on the co-construction of the experience, both in-context and across time and space (Home and Jary, 2004; Elliott et al., 2020).

The objective of this study was to explore the conceptual contrasts athletes made between motivation and perceptions of agency, as constrained and enabled by structural parameters, in-context and across sport experiences that compounded to the athlete's youth career (Home and Jary, 2004; Rose, 2006). The implications of the study center on exploring the potential a constructivist-based theoretical framework holds in expanding how youth sport participation patterns are examined and conceptualized. The following research questions guided study design and data analysis:

- What structural factors supported continuation in a sport environment?
- What personal (micro-level) factors supported continuation in a sport environment?
- How did structural and personal factors compound across time and contexts to affect sustained participation in youth sport?

### Situating Theory in Youth Sport Participation Trends

The predominance of research on supporting youth sport participation has centered on youths' motivation to participate in sport and the perceived benefits of participation. Among the primary motives to participate that youth identify are: (a) to have fun, (b) to be with friends, (c) to get exercise, and (d) for the excitement or challenge of competition (Fraser-Thomas et al., 2005; Balish et al., 2014; Wendling et al., 2018). Youth's motives for sport participation were derived from analyses primarily grounded in theory that is situated in a post-positive paradigm, such as Ryan and Deci's (2000) Self Determination Theory (SDT) of motivation and personality, in which micro-level processes are quantifiable determinants of motivation (Balaguer et al., 2017; Gould, 2019).

Through the lens of SDT, motivation is predicated on the level to which the three basal human needs of autonomy, competence, and relatedness are met in a given context, consequentially affecting the degree to which behavior is self-determined (Ryan and Deci, 2000, 2002). Intrinsic motivation and basic need fulfillment—the foundational tenets of SDT—are widely supported as determinants of youth's motivation to participate in sport (Balaguer et al., 2017). Though work framed in motivational theory has made a significant and well-supported theoretical contribution to examining patterns of participation in sport, analyses grounded in a post-positivist lens might be limited in providing a holistic framework for examining youths' compounding experiences within and across environments as co-constructed (Home and Jary, 2004; Bateman et al., 2020; Reverberi et al., 2020); SDT identifies social contexts as influences, but it does not account for the effects of perceived agency or positioning in-context, or the amalgamation of experiences an athlete gains across multiple environments as they age through the youth sport system (Home and Jary, 2004; Balaguer et al., 2017). Motivational lenses are critical to examining sustained participation among youth, but tend to operate in a single context, delimiting analyses around motivation to those context-specific factors (Petitpas et al., 2005; Balish et al., 2014; Crane and Temple, 2015).

#### Structuration Theory

Giddens' (1984) Structuration Theory (ST) is a macro social theory grounded in a constructivist lens that provides for the degree of influence the interaction between an individual and their environment(s) has across time and space. ST is often associated with organizational research grounded in institutional theory (Scott, 2001; Veliquette, 2013); very little research on sport participation and sport environments has been conducted through the lens of ST despite the presence of sport research framed in constructivist paradigm, which is common in investigations designed around exploring or interpreting the meaning ascribed to sport experiences (Beni et al., 2017). ST has been employed as the theoretical lens in research conducted by Cooky (2009), who explored girls' constructions of interest in sport, by Ogden and Rose (2005), who explored African American youth's orientation toward baseball participation, and Dixon (2011), who examined the social construction of football fandom. Though each of these sport-related research papers have garnered attention, reflected in their citation indices, an ST lens does not otherwise appear in the body of knowledge on participation patterns or lived sport experiences.

An application of ST to a sport context provides further opportunities for insight into understanding the individual's position within an environment, how norms and rules impact the individual, and the influence of prior experiences across age levels and contexts, which Cooky (2009) and Ogden and Rose (2005) found to be key determinants of participation patterns in sport. The foundational constructs of ST are agency and structure, and the recursive relationship between them, in which micro (agent) and meso (structure) level activities enable and reproduce the other (Giddens, 1984; Home and Jary, 2004).

##### Agency

Agency, opposed paradigmatically to motivation, encompasses the individual's opportunity to make decisions, extending boundaries of motivation to include the capacity to act, which is affected by the organization of power in the context (Giddens, 1984). The term agency implies the “power of effect” (Giddens, 1984, p. 41), and so is not determined by the intent or outcome of an act, but that the individual is the perpetrator of the act. Thus, the construct is grounded in the capacity of the individual to have chosen a different act (or none at all) at any time. According to Giddens (1984):

To be able to act otherwise means being able to intervene in the world, or to refrain from intervention, with the effect of influencing a specific process. An agent employs a range of causal powers, including influencing others. Action depends on the capability of the individual to make a difference to a pre-existing state of affairs or course of events. An agent loses the capacity to do so if he/she can't exercise power. (p. 46)

Agency, as a construct, has reach beyond the individual's intended outcome (relative to the self), extending to an impact on the environment in which the act is chosen. This is particularly salient in the youth sport environment, as the athlete's choice in behavior is not fixed or conclusive—the athlete's experience extends across time; athletes learn adaptive behaviors and interpersonal skills across varying contexts (e.g., sports, teams, relationships; Home and Jary, 2004; Cooky, 2009; Reverberi et al., 2020).

Agency does not occur in a fixed time and space, but is a function of “reflexive monitoring” (Giddens, 1984, p. 43). Decision-making is not only based on the agent's evaluation of their own behaviors, but is also based an evaluation of the behaviors of other actors in the environment, and the subsequent consequences (Giddens, 1984); decisions are grounded in compounded observations of consequences within and across environments. Giddens (1984) distinguishes between intended and unintended consequences of an individual's actions across time and space, where intended consequences result in a perceived causal impact on the environment, in contrast to SDT's autonomous locus of causality (Giddens, 1984; Ryan and Deci, 2000; Home and Jary, 2004; Rose, 2006).

##### Structure

Structure is the second construct central to ST. Structure is defined as the contextual organized rules and resources that enable individual agent's actions (Giddens, 1984). In any structure, the rules and the socialized norms for behavior provide the framework in which agency is constituted. Rules are guided by structural common practice, or routines, by which actors are enabled to make decisions, as well as demonstrate an understanding of the normative behaviors within the structure, reconstituting the routine (Veliquette, 2013; Oppong, 2014). Where rules dictate the range of possible decisions or behaviors, resources are the means through which an agent performs an act (Giddens, 1984).

The recursive nature of the relationship between agency and structure is such that positioned agents influence the structure, but it is the structure that constitutes the agent's range of possible decisions and behaviors (Giddens, 1984). As agents act in a given structure, they move the existing parameters, recreating or reproducing the structure over time. This “Duality of Structure” (Giddens, 1984, p. 47) is the foundational tenet of ST, and represents the critical contribution ST can make to an examination of sport environments through a constructivist lens.

According to Giddens (1984), resources within a structure are either allocative or authoritative. Allocative resources are those that are tangible—control of material goods or access to employ material goods, and thus are particularly salient for low SES individuals or families (Cooky, 2009; Veliquette, 2013). Examples of allocative resources in a sport context are the opportunity and access to participate (e.g., finances, location, and offerings), or the perceived competence of the agent within the team or competitive structure. Allocative resources are any holding that enables the agent to influence the current practice within the structure. Authoritative resources are less tangible, such as power or platform, and reflect the capacity of the actor to employ allocative resources to transform the structure (Giddens, 1984; Veliquette, 2013; Oppong, 2014). The recursive nature of agency and structure is predicated on authoritative resources, as it is the capacity to influence the structure that results in the reconstitution of it (Giddens, 1984). However, if the agent does not have the allocative resources required to support authoritative capacity, influence is not possible, which is referred to by Giddens as “positioning” (Giddens, 1984, p. 109).

The positioning of an actor is determined by the actor's access to and employment of resources as constrained by structural parameters (Giddens, 1984; Cooky, 2009). Individuals within a structure will be uniquely positioned based on their contrasting levels of allocative and authoritative resources. It is this balance (or imbalance) of distribution of resources that both enables and constrains the agent's potential for influence on the structure (Giddens, 1984; Rose, 2006). Thus, structures are comprised of the individuals within them, and are reproduced by the interplay between agents and the resources they hold.

## Methods

By the nature of ST as a macro social theory, the examination of sustained youth sport participation through this lens is situated in a constructivist paradigm that supports positioning the study within a greater societal context (Cooky, 2009; Kamal, 2019). In a constructivist paradigm, an individual's accounts of lived experiences, and the researcher's interpretation of those accounts, are epistemologically situated as socially co-constructed (Kamal, 2019). Constructivism is foundationally commensurate with Giddens (1984) ST, as the theory is predicated on the interplay between structure and the agent's interpretation of consequences and positioning within the structure (Vaismoradi et al., 2013; Veliquette, 2013; Kamal, 2019). Additionally, the paradigmatic contrast embedded in the design of this study positions the researcher epistemologically to interpret and analyze participants' accounts of their experiences across their youth sport career (Kamal, 2019).

A deductive thematic analysis situated within a constructivist paradigm was the qualitative method employed to collect, analyze, and frame the discussion and reporting of the data. A thematic analysis methodology is on the simplistic end of the interpretive continuum of qualitative research methods, where discussion of the data is composed of descriptions of participant accounts; in contrast, and opposite an interpretive phenomenology, in which author interpretations comprise the data discussion (Vaismoradi et al., 2013). Thematic analysis supports developing nuance in a particular dimension of a story or a specified group of themes (Braun and Clarke, 2006; Vaismoradi et al., 2013); in a thematic analysis, patterns are identified and extracted from narratives of life stories and experiences collected around a specific set of phenomena (Vaismoradi et al., 2013). Data analysis in this methodology is conducted through segmenting and describing patterns in the data (Vaismoradi et al., 2013). Patterns in the data that substantiated the themes in this study were derived from inclusive coding of data segments, which, per Braun and Clarke (2006) supports the rotation of coded segments across different themes to develop richer context around the data.

### Methodological Rigor

Issues of credibility, dependability, and confirmability were addressed through a comprehensive and transparent audit trail, the constant comparative method in data collection and analysis, triangulation across theoretical lenses, and examination of negative cases (Zhang and Wildemuth, 2009; Creswell, 2012). Through the process of coding the data, the support for deduced themes was recorded and developed in the audit trail (Zhang and Wildemuth, 2009; Creswell, 2012). The constant comparative method was used to systematically compare existing codes, emergent sub-themes, and to categorize units of text that composed patterns in the data that substantiated the themes (Zhang and Wildemuth, 2009).

Two methods of triangulation supported data analysis: (a) theme saturation across multiple sources (interviewees) and (b) across theoretical frameworks from which the themes were deduced (Zhang and Wildemuth, 2009; Vaismoradi et al., 2013). Trustworthiness was supported through two peer-debrief sessions over the coded data and appropriateness of themes, and through an examination of negative cases (Creswell, 2012; Vaismoradi et al., 2013). The examination of negative cases was critical to data analysis, as the comparisons that participants drew between *those environments that supported participation* and *those environments that did not* were central to informing the discussion of results and potential implications of the study.

### Design

The authors were granted approval by their institutional review board to interview the participants. The data were collected through semi-structured interviews that were conducted on-site at IMG Academy. The interview guide was generated through a review of research on youth sport participation patterns, theory employed in analyses around motivation and supportive frameworks, and the constructs of ST that are salient to the research questions. Interviews were conducted with individual players in a private setting over the course of 3 weeks, after 2 weeks of the interviewer's immersion in the environment.

The interview guide consisted of three primary sections, organized by the age at which participation occurred: elementary school, middle school, and high school. Participants were prompted through their account of experiences at each age level, chronologically examining sports played, teams, social influences, coaches, and parental involvement. For instance, participants were asked “What did you like most about the sport?” and “Tell me about your team.” Participants were also asked for their interpretations of their role(s) on the team (e.g., on-field and off-field), and for their recalled perceptions of their relationships with coaches. During each interview, if the participant identified a particularly salient memory, probing follow-up questions were posed to the participants to engage them in in-depth conversation to further explore the impact of the event (Creswell, 2012). The interviews ranged from 44 to 73 min and could be characterized as a conversation with the participants about their interpretation of the most poignant memories from their youth careers, while layering in structural components, such as rules and norms. A total of 1,254 min of transcribed interview data comprised the data set.

#### Participants

Purposive sampling identified those who could inform the study with their experiences in youth sport (Zhang and Wildemuth, 2009; Vaismoradi et al., 2013). The participants (*N* = 20) were collegiate soccer players from universities across the US, representing NAIA (*n* = 1), NCAA Division I (*n* = 17), and NCAA Division II (*n* = 2) men's programs, and included three international participants who had experience in non-US youth sport systems (*n* = 3).To reach theme saturation, 20 interviews were conducted with men ages 18 (*n* = 1), 19 (*n* = 4), 20 (*n* = 9), 21 (*n* = 5), and 22 (*n* = 1). The participant pool was comprised of those who identified as Hispanic/white (*n* = 2), Hispanic/Black (*n* = 1), Hispanic with no racialized identification (*n* = 2), Black (*n* = 1), white (*n* = 14). The participants in the sample represented success stories that were anchored by access and opportunity, where *success* was defined as having sustained participation in organized competitive sport beyond the youth level.

#### Procedure

The interviews were conducted by the first author, whose experiences in soccer established commonality in the language of the sport and supported the interpretation of the athletes' accounts of the experiences and the development of the codebook (Attride-Stirling, 2001). For example, when a player recalled being moved from right winger to right back when he progressed from his club team to the US Soccer Development Academy, there was a mutual understanding of the position and competitive level of the moves; knowledge of the game enabled the first author to translate and interpret the account beyond the manifest meaning of the moves, engendering an exploration of the athlete's perception of the moves and the implications the moves had on his youth experience. To maintain confidentiality in reporting, details in datum that might be identifiable were omitted entirely or obscured in cases where the nuance in the datum represented a salient expression (Vaismoradi et al., 2013). Each player was assigned a pseudonym in reporting.

Prior to data analysis, *post-hoc* notes on the interviews were detailed in the audit trail. The interview notes evidenced initial interpretations of each interview and amendments to the interview guide. The audit trail also contained notes on previous research, demonstrating the origination of the established themes, and the appropriateness of the codes based on initial interpretations of anecdotes provided in the interviews. The record of how the codes were generated and of the critical analysis of the interviews prior to data analysis addressed issues of credibility in the study (Zhang and Wildemuth, 2009).

A deductive thematic analysis was conducted on the interview data, which were transcribed by a third party. The first author, who had extensive experience in the sport, applied the themes to the data and maintained the codebook, as constrained by the apriori themes, that emerged from data analysis. The themes were identified deductively, delimiting data analysis, due to the saturation of research framed in SDT and complementary theoretical frames situated in a post-positivist motivation-oriented lens, such as Achievement Goal Theory or Expectancy Value Theory, which have shaped the body of knowledge on youth sport participation (Balaguer et al., 2017); little research has applied the agent/structure-level operations of ST to sustaining sport participation (excluding the previously identified authors), necessitating a starting point for construct development and initial measures of analysis framed in ST and a constructivist lens (Braun and Clarke, 2006; Vaismoradi et al., 2013). The themes identified from the literature on sport participation and attrition patterns were: (a) fun and enjoyment, (b) individual impact on the environment, (c) the environmental structure, (d) peer and coach relationships, and (e) parent involvement.

Coding the data was an iterative process that initiated during interview as researcher notes on initial interpretations of critical components of the story (Attride-Stirling, 2001), such as *politics*, a code that emerged under the *impact on environment* theme. The codebook was kept by hand in an audit trail in which codes were documented, revised (constant comparative), and organized through the progression of the interviews and subsequently through data analysis (Zhang and Wildemuth, 2009; Creswell, 2012). Analytic software was not used to code the data. The data were analyzed through labeling text segments in word doc transcriptions and the segments were pasted into excel spreadsheets tabbed by code and organized by theme/subtheme.

The draft of the codebook generated from the interviews provided an initial coding frame through which the transcribed data were initially analyzed (Attride-Stirling, 2001). Through iterations of data analysis, the codebook was refined and reorganized to incorporate additional codes or emerging subthemes (Attride-Stirling, 2001).

## Results and Discussion

Data analysis supported the themes identified (deductively) through a review of the extant literature on youth sport participation (as framed in motivational theory) and a review of ST, which does not have a presence in the literature on youth sport participation (Braun and Clarke, 2006). The themes (a) fun and enjoyment, (b) individual impact on the environment, (c) the environmental structure, (d) peer and coach relationships, and (e) parent involvement were each substantiated in categorizing the common factors that evidenced to affect continued sport participation. However, based on the data from the participants, the thematic structure was amended to promote the themes *individual impact on the environment* and *environmental structure* as the two dimensions of the data that were most associated with the design of the study (Attride-Stirling, 2001; Braun and Clarke, 2006).

The themes *peer and coach relationships* and *parental involvement* are not reported independently; the data and codes associated with *peer and coach relationships* were able to be categorized under the two critical themes and the data and codes associated with *parental involvement* did not represent a salient dimension in the data, beyond access, which is an assumption of study design. The data and codes associated with the deduced theme *fun and enjoyment* are developed in another manuscript because a distinction in meaning between the concepts emerged from the data, and thus the authors determined that the micro-level process warranted a rich description.

Through a constructivist lens, and in accordance with ST, the theme *impact on the environment* (i.e., bottom-up agency) evidenced to be inter-related to the theme *environmental structure* (i.e., top-down structure; Giddens, 1984); the two critical themes are not reported here as exclusive. Though in this study it is assumed that agency and structure are inherently inter-related as constructs, for the purpose of exploring stratifying the ST constructs, participants' reports of individual impact on the team or coach were coded under the *individual impact on the environment* theme, and their interpretation of the rules, norms, and expectations imposed were coded under the *environmental structure* theme.

### Theme: Individual Impact on the Environment

The participants consistently characterized *impact* within a context as a function of allocative and authoritative resources, which configured to determinants of sustained participation across contexts (Rose, 2006; Veliquette, 2013). The participants' perception of impact on the environment was primarily associated with two antecedents: competence, which demonstrated as the most salient allocative resource, and the opportunity to demonstrate competence, which directly aligned with authoritative resources as a mechanism (Kabeer, 1999; Veliquette, 2013). The *individual impact on the environment* theme was partitioned into two subthemes: *impact on play* and *impact on structure* (see Table 1).

**Table 1:** Exemplar data segments characterizing the patterns in the Individual Impact on Environment theme.

**Theme: Individual Impact on Environment**	
Impact on Play	Jay: “I felt that I was better than the kids and I felt that I was here, at the big-time club and I can actually play. I was good at it and that is what made me work hard and made me feel in my mind that I was better than some of the kids who were playing… So, being good, it made me…I liked it.”
	Oliver: “In basketball I felt like I wasn't as big a part of the game, there wasn't as much meaning, just running back and forth and throwing the ball. With soccer, I could get the ball, I could score, I could pass, I could do all this stuff. I enjoyed it more.”
	Ryan: “In football I wasn't like a quarterback, so I wasn't leading the team or touching it all the time. I felt like I didn't have as much of an impact, so I just didn't like it.”
	Willis: “There would be games where we wouldn't have been in the game if I wasn't in goal. You can tell when everything is really easy for you, when the shots are easy, play is really easy. It was really easy for me. The shots were not challenging, the training was not challenging, the games were not challenging. I could do what I want.”
Impact on Structure	Domingo: “We just understood one another and if I would disagree with him, we would argue. It wasn't like a brotherly relationship because he was older than me but we understood there was respect there so I was never going to state something that was out of line to him but I would voice my opinion and we just understood each other. In my mind I could tell him what I think. He understands that I am a good player, I can do both things and I won't go against what he says. I just might not agree with it.”
	Edwin: “One of the reasons I left was the head coach was just a jerk. He would promise playtime to people and then bench them. He would tell them they were doing well and then not play them. He played his favorites game hard. Those players who stuck around from the [former team] were put on a pedestal and it was hard to knock them off.”
	Willis: “I felt like we were isolated because we weren't in the group. But I knew I could play with those kids. So that sucked that I didn't. I felt like I wasn't getting the opportunity I deserved. The coaches would focus on the best team, the A team.”
	Oscar: “So, it was a way to make me step up into a leadership role and take on the responsibility. It was, all right, I am an example, so now I have to play well, I have to push myself harder so that everyone else knows that if I am trying harder, then they should be trying harder too. So, I think I was definitely an impact player on that team. I think that by the time I was a junior and a senior I was one of 2 or 3 guys on the team who the coaches really looked to as the leaders on the team.”

#### Impact on Play

The impact that participants perceived in what they characterized as positive youth environments was notably different from the level of impact they perceived in negative environments. Positive youth environments evidenced to be those in which the participants perceived competence in conjunction with the opportunity to leverage their ability in their performances. At the younger age levels, the participants did not continue in sport environments in which they did not possess competence, or lacked allocative resources. However, in soccer, where perceptions of ability were stable, the primary distinction between negative and positive environments was the degree to which the athlete perceived authoritative resources (Kabeer, 1999).

#### Resources

The participants identified that they perceived an impact on their team, or structure, across sports and developmental stages. Although perceptions of *impact* were coupled with perceptions of competence, the distinguishing factor between the two constructs was the player's opportunity to demonstrate competence. In positive youth environments, the manifest perception of competence was evidenced in the participants' belief that they were “*a good player*.” The latent presumption in their accounts of impact was that they had the opportunity to demonstrate their skill. Thus, the participants invariably perceived that they possessed both allocative and authoritative resources in positive environments (Kabeer, 1999; Veliquette, 2013). Ryan described perceiving both types of resources through his account of his role as a skilled player and leader:

“I was kind of like a point guard and somewhat the leader of the team. I was kind of the best player, so my friends just kind of gave the ball to me. I was always the one organizing things, I was like that in every sport that I played.”

#### Freedom

Positive youth environments supported the players in their expression of skill and ability, or provided them with the authoritative resources to demonstrate allocative resources (Kabeer, 1999; Amorose and Anderson-Butcher, 2015); a secondary component of impact was the perception that the player had the freedom to play unrestricted. The term *freedom* was frequently employed when participants spoke to the contextual set of rules or practices imposed by the coach. Within the coach's framework, the participants valued the opportunity to make their own decisions.

A sense of freedom to play unrestricted was a function of perceptions of competence, as the participants interpreted *freedom* as an opportunity to demonstrate their skill. Oscar differentiated the sense of freedom he felt between two different types of imposed structure under the command of two different coaches:

“I never really did that with [Coach 1] because his style was more of a strict back 4, if you were in the back you stayed back, not so much freedom to go up. But [Coach 2] was a lot better about letting his outside backs to go up and getting involved in the play and letting them be creative when they needed to be. I think that helped a lot. I remember that when I got recruited to play soccer in college, one of the main reasons was they saw me score a goal from the left back position and that would not have happened if I stayed with [Coach 1], obviously. Giving me that freedom and support to have the confidence to go up and be creative and do what my Dad called ‘a little bit of magic on the ball’ was really important.”

A paradigmatic delineation between agency and motivation was most effectively elucidated by the participants' accounts of negative experiences. Though the freedom to play was indistinguishable from perceptions of autonomy in accounts of positive environments, descriptions of negative environments often reflected that, even if the participant was the source of their behavior, where the behavior was not allowed, or was not supported by the environmental structure, they did not perceive freedom. As Oliver explained, autonomy may be perceived in a structure, but without authoritative resource holdings, control of the experience deterred sustained participation in the organized structure (Kabeer, 1999; Amorose and Anderson-Butcher, 2015):

“[Hockey] was more like teaching us certain ways to skate, how to pass different, how to shoot instead of tactical stuff. I just remember that once I got there, I liked playing pick-up and playing in my driveway more. I didn't feel the freedom of it anymore. I felt restricted. It was too organized for me.”

The contrast the participants drew between perceiving competence and the opportunity to demonstrate that competence was linked directly to the rules in-context, supporting agency as a multi-dimensional construct that might more inclusively explain effects of the cultural shift toward structured play among youth and the associated impacts on participation trends (Aspen Institute, 2019).

#### Impact on Structure

In environments in which the players perceived impact on the structure, their interpersonal relationship with the coach was frequently reported to support a dyadic exchange (Rose, 2006; Amorose and Anderson-Butcher, 2015; Reverberi et al., 2020). The relationships that supported participant impact on the environment were frequently characterized by a mutual respect between the player and coach, and a perceived value that the coach placed on the participant's input. The participants reported that they felt empowered to voice an opinion and sometimes even operated as an interpreter for the coach in his (all male coaches were reported) communication with the team. As James described, the participants' relationships between teammates and coach demonstrated as inter-related in-context based on the positioning of the athlete (the relationship between peer dynamics and agency in-context was not clearly delineated through interview data):

“He had a picture in his mind of what he wanted, and my job was to take what picture he wanted and put words to it. Make it easy for everyone else to understand. He would describe and I would always be in that central role. And I would be like, ‘OK, you step, and I will be right behind you and you go to the ball’ and all that stuff.”

Where agency was supported by the coach in what participants described as positive environments, many of the negative environments that were characterized by poor relationships with the coach engaged the idea of the coach “playing favorites.” When the participant perceived that he was not valued by the coach, perceptions of agency and impact were reduced, consequently limiting the belief that his actions could produce outcomes (Cooky, 2009; Oppong, 2014). Darren described that he had considered quitting soccer altogether because he felt that the coach did not value him, despite feeling that he possessed the ability to perform:

“I was getting promised stuff and I was showing the results but [he] didn't believe in me. I wasn't one of his favorites. I knew I was good enough to play on the team, he just wouldn't give me the chance. At that time, I was really thinking about stopping soccer.”

In addition to the strains on the coach-player relationship that participants perceived in negative environments, limitations on opportunity imposed by the environmental structure of an organization also impeded the participants' perception of authoritative resources (Kabeer, 1999; Sheerin et al., 2020). Often, in characterizations of negative environments, the participants identified that they were not afforded the opportunity to demonstrate their ability by forces that extended beyond the immediate context to include broader positioning of the team or player. Ryan explained how his perceived lack of authoritative resources was shaped by the organization of power in-context, including the coach, but extending to the structuring of the competition:

“That was one of the reasons that I quit, I just found it to be so political. One of the most frustrating things I had to deal with was when I was on the ODP team: I played the game and thought I played really well, but a few other kids did too, but there was one kid who didn't play, didn't play at all that game and our coach picked him to go to the regional game because he was resting him for the region pool game that night so he would play well.”

Pervasive throughout the common accounts of negative experiences, the most significant detriment to the participants' perception of impact was a lack of authoritative resources. Giddens (1984) defines authoritative resources as “types of transformative capacity generating command over persons or actors” (p. 63). Giddens (1984) also qualifies that “allocative resources cannot be developed without the transmutation of authoritative resources” (p. 269). As evidenced in the participants' accounts of environments in which they perceived both allocative and authoritative resources, the environment was perceived as positive or beneficial. Conversely, though the participants reported that they possessed the necessary allocative resources (competence) across contexts, negative environments were those in which they did not possess the necessary authoritative resources to develop or demonstrate their abilities (Sheerin et al., 2020).

### Theme: Environmental Structure

Participants' accounts of the coach's expectations or environmental parameters were coded under the theme *environmental structure* (top-down structure). The participants' accounts of their youth environments evidenced significant variance across contexts within each individual's career; each participant reported being exposed to multiple structures, both positive and negative, which were invariably characterized by the coach's personality or coaching style (see Table 2).

**Table 2:** Exemplar data segments characterizing the patterns in the Environmental Structure theme.

**Theme: Environmental Structure**	
Coach Demands	Damon: “The other thing was their coaching style was harsh like my old coach, but they didn't really care as much individually. I wasn't getting as much attention and I wasn't used to that and I didn't know how to deal with not getting the attention I had gotten since I was 11. I could deal with the harsh coaching when I was getting the attention before but now that it was like that, without it, I was getting pissed off.”
	Oscar: “He wasn't afraid to get into you. He was always really clear about what he expected from everyone. If you weren't meeting it, he would tell you and would tell you how to fix it. He would not just say, ‘you’re not doing this, do it better'. He would say, ‘this is what you need to do now I will show you how to do it’. ‘If this happens, you need to do this, you need to be thinking about this’.”
	Willis: “He knew what to say and how to say it. Sometimes you don't say something to a player, you allow him to figure it out on his own because you know he can. Whereas other times they have done the same thing several times and you say hey, do this or don't do that. During games and in practice, he knew when to let us work it out as a team internally and when to voice his opinion. I think he balanced that really well.”
	Ryan: “Screaming. Whenever he was mad, say we had played a bad half, we would go into the locker room and we would know it was coming. He would come in and knock over the water, punch a locker and scream at us. If it was going good, he would relax but most of the time he was intense. It was never, hey you messed that up, next time you need to do this. It was more like, you seriously messed this up, what are you doing? We have worked on this before, how can you not get it?”
Coach Expectations	Ira: “Easy, they were really clear on what they wanted, where some other coaches were more emotional rather than clearly laying out what they want. I clearly knew the system, what he was looking for, everyone knew their roles, style of play. All really level-headed, nothing ever got to him really, ever.”
	Brian: “The new training was good, it got much more intense. This new coach, who I am still really close to today, he demanded a lot more, which was good. It wasn't too big of a jump, but you could tell that the demands he made of some kids was a lot, some kids couldn't handle it and some kids could. He was the guy who really pushed me, he was the first person who I met in soccer who was going to really be hard on me, wouldn't let things slip. If he wanted me to do something different, he would let me know, real stern. There were a few times in practice where I would cry.”
	Damon: “If it was a big game, he would be positive, not on your back. But if it was a lesser team and we were messing up, he would get really pissed off, shouting and stuff. We played a bunch of big games, State cups and national games and those games it was like nothing negative. Because I think he didn't want us to crack under pressure. But when it came to a bad team we were playing and we started messing around he would get really pissed off.”
	Ryan: “I was probably the closest one to him because again I was kind of like the leader on that team. If he ever needed anything, he would come to me and say make sure this gets changed on the team, or if someone needed to change their attitude, he would come to me and tell me to talk to this kid and help him out.”

The two dimensions of how environmental structure was imposed, and how that impacted the valence of an environment, were coach demands and coach expectations. Without clarity or consistency in expectations, the players were not able to navigate the imposed structure, which evidenced to detract from continuation; however, the opportunity to move into alternative environments supported continuation.

#### Coach Demands

The demand for skill execution was consistent across the participants' assessments of positive environments. However, the manner in which the demands were communicated by the coach was a determining factor in individual assessments of the environment as positive or negative (Witt and Dangi, 2018; Howie et al., 2020). Intuitively, environments in which the coach communicated demands exclusively through only broad or negative language, without providing positive feedback or information, detracted from player engagement. The participants were accepting of those coaches who communicated demands negatively if positive support and encouragement or information were provided. Damon described a coaching style that he remembered as demanding but encouraging, which supported continuation:

“He was kind of like a weird mix between super nice and caring and always trying to pick up you up and be there if you failed, and at the same time he was really hard on you. When we were pretty young, he would be yelling at you if you made mistakes and at the same time, if you lose a game or don't make a team he would pick you up and be like, don't worry about it, you are a good player.”

Several participants characterized negative environments as those in which the coach's demands were perceived to be unrealistic, or where the coach did not accompany demands with positive feedback or actual information. Though elevated demands were commonly assessed as contributing to development, the participants' ability to interpret and endure imposed demands evidenced as variable across individuals. Environments in which the participants felt that they were unprepared to mitigate the imposed demands, or were not provided sufficient feedback to do so, evidenced to detract from their sense of agency. Willis described a negative environment that was characterized as such because he felt that the demands were unrealistic:

“They were all very intense, it didn't matter that we were in 6th grade, it didn't matter that I was 12 or 13 and had to go up against an 8th grader. That's how they did it, you are young, you have got to get bigger so you are going to go up against the biggest kids. That didn't work for me.”

As evidenced by the participants' reports, at some point in their youth career, they were each subjected to an environment in which the coach was “a yeller,” or in which elevated demands were placed on them. However, these characteristics were not frequently associated with negative environments. Conversely, yelling and elevated demands were often associated with positive environments, if the demands were accompanied by positive support and care or simply information that enabled the participant to meet the demands.

#### Coach Expectations

Though participants reported that coach behaviors that would commonly be accepted as negative (i.e., yelling or placing excessive demands on the players) were not associated with negative environments, the coach's expectations were consistently reported as a determinant of the valence of the environment. The significance of the coach's expectations is closely aligned with player's perception of impact on the environment (developed earlier), as the expectations are a manifestation of the environmental structure, and the player's agency within the structure is delimited by the imposed parameters (Home and Jary, 2004; Cooky, 2009). Consequently, the participants identified that clear and predictable expectations, and role definition, were elements common to positive environments, which supported their perceived impact on the structure.

Oscar explained how a coach who constructed a positive environment provided role clarity for him, which was central to his ability to navigate the structure:

“He was always really clear about what he expected from everyone. If you weren't meeting it, he would tell you and would tell you how to fix it. He would not just say, ‘you’re not doing this, do it better'. He would say, ‘this is what you need to do now I will show you how to do it’. ‘If this happens, you need to do this, you need to be thinking about this’.”

The athletes' descriptions of environments that supported participation were focused on their learning to meet expectations through the provision of specific and informational feedback, which would operationalize (in-context) an allocative resource; where the environment was positive without demands or information, it was not sustainable, but in contexts in which excessive demands were perceived in conjunction with specific information to meet those demands, participants reported that they engaged with the team and sport more completely.

In accordance with ST, the participants identified that positive environments were those in which the expectations were clear and predictable, and the expectations included the information necessary to meet demands. However, the participants' appreciation of clarity in expectations was more effectively demonstrated through their accounts of negative environments, in which participants' described coach expectations as unclear or inconsistent. Also in accordance with ST, participants characterized negative environments as those in which their inability to define or predict expectations affected their ability to make constructive or routined decisions (Giddens, 1984; Rose, 2006). Ira described an example that demonstrated why he felt unprepared to interpret the coach's expectations even in a familiar context/relationship:

“Another would get praised for something and you wouldn't. There was a different expectation. I remember there would be times where I would whip in a great cross, and nothing would be said of it. And then, another guy he liked would whip in a great cross, and he would get excited, he would be very charismatic about it. I don't know if it was just because he knew me forever, or if that was just what was expected. There would be an expectation to put the ball in the box and I would do it 5 times out of 5, then the 6th time mess up. He would get on me. Another guy got it 1 out of 6 and he gets praised.”

The participants' reports of negative environmental structures mirrored their reports of limitations in their impact on the environment. In those environments in which the players perceived a significant impact on the environment, their perceptions of competence, or allocative resources, were coupled with their ability to demonstrate competence, or authoritative resources (Giddens, 1984). Due to the recursive nature of the relationship between agency and structure, when the parameters in the structure were constantly moving, the agent was unable to discern the appropriate decision or course of action, which undermined their perception of the resources they possessed (Giddens, 1984; Kabeer, 1999). Consequently, unpredictability or inconsistency in the coach's expectations was a determining factor in the participants' assessment of negative environmental structures; the players expressed that they needed to know the expectations in order to meet them.

## Conclusions

The nature of sustained participation, or keeping kids in sport, is such that the motivation to participate is a dynamic process that evolves as the athlete ages and gains experiences (Home and Jary, 2004; Amorose and Anderson-Butcher, 2015). Where the predominance of extant literature on youth sport participation and attrition patterns is situated in a post-positivist paradigmatic lens, often operating in a snap-shot (Balaguer et al., 2017), the purpose of the study was to explore the potential a constructivist-based theoretical framework holds in identifying novel conceptualizations of those factors that supported sustained participation; in other words, in what ways were the success stories co-constructed through the players' progression in soccer in and across contexts? Through this examination of sustained participation, which centered the agency/structure relationship in-context and across the youth sport career, participants described the central elements that might be explored in order to extend the literature on supporting continued youth sport participation. It is not suggested here that ST be considered as an explanation for motivation in sport participation, but that ST can provide a lens through which sustained participation across time and space can be evaluated holistically through a constructivist lens.

The data collected to address the research questions that guided this study, around the compounding of personal and structural factors, demonstrated that a critical function of sustained participation reflected the fundamental tenet of ST: that an agent positioned with allocative and authoritative resources can affect the structure (Giddens, 1984). In this study, the participant's perception of authoritative resource holdings was the most salient element of his experiences across time, where authoritative resources were described in terms of the participant's reported impact on play and their reported impact on the group, or the structure.

Where the micro-level element of positive experiences was the perceived holding of authoritative resources, the paramount structural elements that evidenced to support continuation were coach demands and coach expectations, the subthemes of the *Environmental Structure* theme. The subthemes are differentiated as *coach demands* being the delivery of the coaching, where *coach expectations* reflected the formal or informal and normative rules in-context, as imposed by the coach. The opportunity to move environments (i.e., away from those environments that were characterized as negative; into more elite levels of competition), also evidenced as a structural component of sustained participation.

### Implications

The primary contribution the ST lens provided for reframing participation data evidenced to be the concept of the athlete's positioning within a structure, predicated on allocative and authoritative resource holdings (Giddens, 1984). Though allocative resources, such as access and competence, are ubiquitous in extant literature on youth sport participation, authoritative resources (the opportunity to employ allocative resources) within and across environments is a construct that has received less attention. The holding of authoritative resources evidenced to be a critical determinant in the participants' assessments of an environment as positive or negative, where positive environments supported sustained participation and negative environments detracted from the participant's intent to continue. Recursively, the structure of the environment (e.g., rules, norms, and the coach's expectations) dictated the participants' perceptions of holding authoritative resources; environments in which the expectations were clear and consistent, indifferent to the valence of the coach's communication, were identified as positive environments that supported continuation.

A second contribution an ST lens provides in examining sustained participation is the allowance for prior experiences to mitigate negative environments that threatened to demotivate the participants. Though the participants perceived a lack of authoritative resources in singular environments, the perception was not consistent across environments. Respectively, the perception of holding allocative resources was consistent across environments, including those in which the participant did not perceive holding authoritative resources. The participants frequently reported that though they were not granted the opportunity to demonstrate their ability in negative environments, they maintained their perceptions of allocative resources derived from past experiences.

### Limitations and Recommendations for Future Research

The sample of participants in this study presents a limit to the generalizability or transferability of the findings, as each participant represents a success story of those with societal position to access resources required to sustain participation, such as moving to an alternative environment (Schmid et al., 2020). Opportunity and access to participate in sport are limited for many youth (Aspen Institute, 2019), but are assumed for the participants of this study.

Commensurate with the assumption about the study sample, a second limitation of the study is that it operates in the micro-level processes of the relationship between agency and structure as positioned in a constructivist lens, and framed in a macro-level social theory, without positioning the contexts within the greater youth sport system. Though a macro-level analysis of the duality of structure is beyond the scope of this study, as this study examines only micro-level agency as constrained by structural parameters, the position of a structure within a system, and systems as they constitute structuration, should be identified in order to situate the functions of the agent and the structure within ST. While the co-construction of local contexts cannot be decoupled from their position in greater society (Giddens, 1984), the conclusions and implications of this study are constrained by study design that does not position the youth sport contexts examined into greater social systems. Future research on the impact of sport structures on educational systems or social systems is necessary to comprehensively examine a macro-level association with sport participation through the lens of ST.

The participants' perception of holding authoritative resources emerged as a critical determinant of motivation to sustain participation in a specific context, and was the primary factor that distinguished a negative environment from a positive one. Though ST has rarely been employed in the analysis of sport environments, the micro-level operations of ST (i.e., allocative and authoritative resources) as the determinants of an agent's position within the structure evidenced to be a tenable framework to characterize negative and positive environments. Thus, a recommendation for future research is to validate the micro-level concepts of ST as factors that support sport continuation through quantitative or structural methodologies.

## Data Availability Statement

The raw data supporting the conclusions of this article will be made available by the authors, without undue reservation.

## Ethics Statement

The studies involving human participants were reviewed and approved by University of Florida IRB. The patients/participants provided their written informed consent to participate in this study.

## Author Contributions

MF designed and conducted the study. MS provided advising oversight and writing support. All authors contributed to the article and approved the submitted version.

## Conflict of Interest

The authors declare that the research was conducted in the absence of any commercial or financial relationships that could be construed as a potential conflict of interest.
